# The potential impact of ChatGPT/GPT-4 on surgery: will it topple the profession of surgeons?

**DOI:** 10.1097/JS9.0000000000000388

**Published:** 2023-04-11

**Authors:** Kunming Cheng, Zaijie Sun, Yongbin He, Shuqin Gu, Haiyang Wu

**Affiliations:** a Department of Intensive Care Unit, The Second Affiliated Hospital of Zhengzhou University, Zhengzhou, Henan; b Department of Orthopaedic Surgery, Xiangyang Central Hospital, Affiliated Hospital of Hubei University of Arts and Science, Xiangyang; c School of Sports Medicine and Rehabilitation, Beijing Sport University, Beijing; d Clinical College of Neurology, Neurosurgery and Neurorehabilitation, Tianjin Medical University, Tianjin, People’s Republic of China; e Duke Human Vaccine Institute, Duke University Medical Center; f Duke Molecular Physiology Institute, Duke University School of Medicine, Durham, North Carolina, USA

Highlights

- This is the first study to summarize the potential applications of ChatGPT (Generative Pre-trained Transformer)/GPT-4 in the surgical field.
- ChatGPT/GPT-4 is capable of participating in multiple aspects of surgical work, including scientific writing, doctor–patient communication, diagnostic imaging, and patients’ perioperative management.
- ChatGPT/GPT-4 could be a good assistant for surgeons, but it was not possible to topple the profession of surgeons.

*Dear Editor*,

With the rapid developments of artificial intelligence (AI), powerful AI-related technologies have entered many areas of life. Recently, ChatGPT (Generative Pre-trained Transformer), an AI-powered chatbot developed by OpenAI, has created a ruckus throughout the world and hits the international headlines frequently. ChatGPT is a natural language processing model and could generate human-like text, allowing users to obtain answers in an intuitive and conversational way^1^. In the initial release version, ChatGPT is a fine-tuned application based on the GPT-3.5 engine. Over the past few months, multiple studies have demonstrated that ChatGPT/GPT-3.5 have great potential in medical applications, such as providing professional medical advice, identifying top research questions, as well as helping in academic writing^2^. However, many scholars also pointed out that in many cases, the answers from GPT-3.5 were too general and vague and even provided incorrect answers. Meanwhile, another major drawback of GPT-3.5 is its non-capability of inputting a picture.

On 14 March 2023, OpenAI unveiled GPT-4, the latest incarnation of this popular chatbot. Of great concern is that it could accept image and text inputs and produce text outputs. In addition, according to the report from the OpenAI team, ChatGPT/GPT-4 exhibited human-level performance on a variety of professional and academic benchmarks^3^. And even could pass a simulated bar exam with a score around the top 10% of test takers. Nevertheless, to our knowledge, there has been no study to assess the potential impact of ChatGPT, especially GPT-4, on the field of surgery. And also, the rapid development of ChatGPT leads us to ask whether it will topple the profession of surgeons? As shown in Figure 1, we have summarized several potential applications of ChatGPT/GPT-4 in multiple aspects of surgical work. There have been multiple studies that evaluated the important role of ChatGPT in scientific writing, which is just as applicable to the surgical area. Therefore, this study mainly focuses on its value during perioperative management^4^.

**Figure 1:**
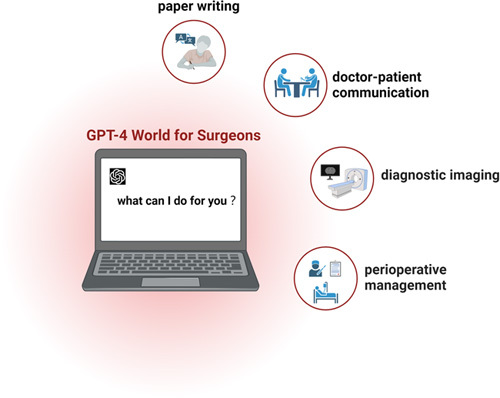
The potential applications of GPT-4 in the surgical field (this figure was created by using BioRender).

Generally speaking, since each patient’s condition, constitution, and recovery capabilities vary, the complex and high-risk decisions made by surgeons under time constraints and uncertainty have a significant impact on patient treatment outcomes. Especially in emergency situations, surgeons often need to make critical decisions in a short period of time. Diagnostic and judgment errors are the second most common cause of preventable harm in surgical patients. Recently, AI technologies, including embodied AI in robotics and techniques like machine learning, are being developed and utilized in the surgical field to assist surgeons in tasks such as diagnosis, treatment planning, and monitoring patient outcomes.

As for ChatGPT/GPT-4, this system may also have greater potential for patients’ preoperative planning, intraoperative decision-making, and postoperative monitoring. For example, ChatGPT could help surgeons collect and analyze patients’ medical history, laboratory findings, and imaging features so as to provide a personalized surgical plan and also assist in surgical simulations to assess the safety and adaptability of operative procedures and predict surgical outcomes. Compared with GPT-3.5, one of the major highlights of GPT-4 is the capacity to handle images. In most cases, apart from clinical examination, surgeons rely on imaging modalities to confirm the diagnosis. Combined with deep learning and big data technology, ChatGPT could help surgeons analyze medical images such as X-ray and computed tomography scans more efficiently and accurately. For example, through well-trained deep learning models, ChatGPT may possess the ability to automatically recognize and segment different structures in images, such as organs, tumors, and blood vessels. Moreover, by extracting and calculating various parameters from medical images, such as tumor volume and the degree of vascular narrowing, it could further help to assess the severity and predict the development trend and prognosis of the disease.

Before surgery, doctor–patient communication, especially preoperative conversation, allows the doctor and patient to establish a trusting relationship, which is essential for the patient to feel confident in the surgical process. ChatGPT could help surgeons communicate more effectively with patients, answer patients’ concerns about surgery, and provide related medical advice. Take the most common appendicitis operation as an example; when we proposed the question that ‘What questions should I ask my patients to make sure they fully understand the risks of appendicitis operation’, the answer is clear and professional (Supplementary File 1, Supplemental Digital Content 1, http://links.lww.com/JS9/A312).

During the intraoperative, it could provide real-time surgical navigation information, including anatomical structures, surgical steps, etc., to help surgeons make more accurate decisions during surgery. In addition, intraoperative monitoring is of paramount importance during surgery. ChatGPT could be a powerful technique that monitors a patient’s physiological parameters in real-time, and once an abnormal situation occurs, alerts surgeons to intervene in a timely manner before there is permanent damage. For regions with insufficient medical resources, ChatGPT could remotely guide local doctors in performing surgeries, improving the quality of the surgery. Furthermore, automatically generating surgical records may become the most popular function for surgeons. While relieving workload simultaneously, key information during the procedure could be recorded, which helps improve medical quality and facilitates subsequent reviews.

In terms of postoperative patient management, other than postoperative condition monitoring, ChatGPT may have possible roles in rehabilitation guidance, pain management, health education, and even follow-up. Take rehabilitation guidance as an example; ChatGPT could develop personalized rehabilitation plans, including exercise rehabilitation, dietary advice, and drug management depending on the different surgery types.

Up to now, AI technology has made significant progress in the field of surgery and has begun to change surgical practices in many ways. Indeed, the emergence of ChatGPT/GPT-4 is expected to bring about a series of advancements in the field of surgery, such as improving diagnostic accuracy, optimizing surgical planning, enhancing surgical efficiency and safety, strengthening postoperative management and rehabilitation, and so on. However, despite ChatGPT’s potential advantages in surgery, there are still many limitations to its current capabilities. In our opinion, ChatGPT still cannot replace the experience and expertise of a skilled surgeon who has years of rigorous training, fine motor skills, and the ability to make complex decisions in real-time. Meanwhile, it is also unable to deal with unexpected situations or complications that may arise during surgery, which requires the surgeon’s agile mind and resilience. In addition, how to use this technique in line with international ethical guidelines is still a topic of debate^5^. Therefore, ChatGPT/GPT-4 is unlikely to topple the profession of surgeons. Instead, it is more likely that AI-related ChatGPT will continue to develop and be incorporated into surgical practice as a tool to assist the work of skilled surgeons. Through working together, ChatGPT and human expertise could ultimately provide better outcomes and improve the overall quality of healthcare. Interestingly, after completing the main text, we tested ChatGPT by asking if this manuscript was suitable for publication in the International Journal of Surgery. Amazingly, it gives an affirmative answer and several professional recommendations (Supplementary File 2, Supplemental Digital Content 2, http://links.lww.com/JS9/A312). Believe it or not, ChatGPT has a certain potential to become an excellent reviewer for journals in the future.

## Ethical approval

This study does not include any individual-level data and thus does not require any ethical approval.

## Sources of funding

There was no funding for this research.

## Author contribution

K.C. and H.W.: conceptualization, methodology, data curation, formal analysis, resources, investigation, and writing – original draft, review, and editing; Z.S.: conceptualization, methodology, data curation, formal analysis, resources, and investigation; Y.H.: data curation, formal analysis, resources, investigation, and writing – original draft, review, and editing; S.G.: conceptualization, methodology, data curation, formal analysis, resources, investigation, and writing – original draft, review, and editing.

## Conflicts of interest disclosure

The authors declare no conflicts of interest.

## Research registration unique identifying number (UIN)

Not applicable.

## Guarantor

Haiyang Wu.

## Data availability statement

The data underlying this article will be shared by the corresponding author on reasonable request.

## Supplementary Material

## References

[1] Castelvecchi D . Are ChatGPT and AlphaCode going to replace programmers? Nature 2022. [published online ahead of print, 8 Dec 2022]. 10.1038/d41586-022-04383-z 36481949

[2] van Dis EAM Bollen J Zuidema W . ChatGPT: five priorities for research. Nature 2023;614:224–226. 36737653 10.1038/d41586-023-00288-7

[3] Sanderson K . GPT-4 is here: what scientists think. Nature 2023. [published online ahead of print, 16 Mar 2023]. 10.1038/d41586-023-00816-5 36928404

[4] Else H . Abstracts written by ChatGPT fool scientists. Nature 2023;613:423. 36635510 10.1038/d41586-023-00056-7

[5] Wang SH . OpenAI – explain why some countries are excluded from ChatGPT. Nature 2023;615:34. 10.1038/d41586-023-00553-9 36854918

