# Supplementary material for: The potential impact of ChatGPT/GPT-4 on surgery: will it topple the profession of surgeons?
Source: Int J Surg. 2023 Apr 11;109(5):1545–7. doi: 10.1097/JS9.0000000000000388 (PMC10389652; doi:10.1097/JS9.0000000000000388)

What questions should I ask my patients to make sure they fully understand the risks of appendicitis operation?


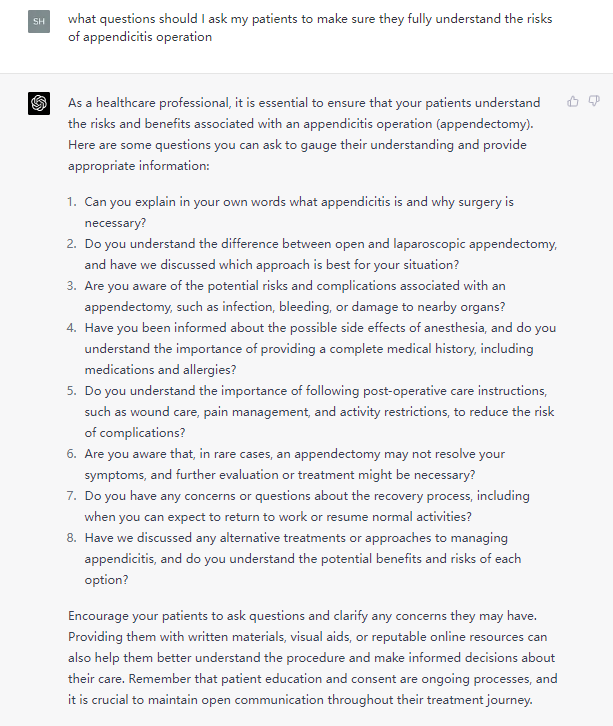

Supplement: Supplementary file 1 [file js9-109-1545-s001.docx]
